# Ablation with the circular pulsed field ablation catheter: Tips & techniques for using the PulseSelect catheter

**DOI:** 10.1007/s10840-026-02436-1

**Published:** 2026-09-15

**Authors:** Gabriel Ballesteros, Sikder M. Zubair Rahim, Faizel Osman

**Affiliations:** 1 Department of Cardiology, Hospital Regional Universitario de Málaga, Málaga, Spain; 2 Department of Cardiology, Hospital Universitario HLA El Ángel de Málaga, Málaga, Spain; 3 Institute for Cardio-Metabolic Medicine, University Hospitals Coventry and Warwickshire NHS Trust, Coventry, UK; 4 Warwick Medical School, University of Warwick, Coventry, UK; 5 Centre for Health & Life Sciences, Coventry University, Coventry, UK

**Keywords:** Pulsed field ablation, Atrial fibrillation ablation, Pulmonary vein isolation, Lesion optimization, Ablation strategy.

## Abstract

Pulsed field ablation (PFA) is a novel method of cardiac ablation in which electrical fields create microscopic pores in cardiomyocyte cell membranes, resulting in cell death through a process of electroporation. Unlike thermal technologies, PFA has preferential cardiomyocyte selectivity, thereby reducing the risk of collateral damage to surrounding structures. The PulseSelect PFA system (Medtronic Inc., Minneapolis, MN, USA) is a 9-electrode circular ablation catheter with a central guidewire used for atrial fibrillation ablation. Currently, there is no consensus on workflow or best practice for this catheter. Its distinctive over-the-wire design and forward-tilted flexible electrode array provide considerable procedural versatility and, in our opinion, may offer advantages in terms of selective lesion delivery, reduced haemolysis, and a lower propensity for coronary vasospasm. Realising these potential benefits, however, requires a thorough understanding of catheter geometry, tissue-contact principles, and manipulation techniques. We present our real-world experience from centres in the UK and Spain, highlighting practical concepts, fluoroscopic and electro-anatomic mapping (EAM) strategies, as well as procedural techniques that may help shorten the learning curve, optimise lesion delivery, and safely expand the use of the catheter beyond pulmonary vein isolation.

**Graphical Abstract:**

**Figure:**
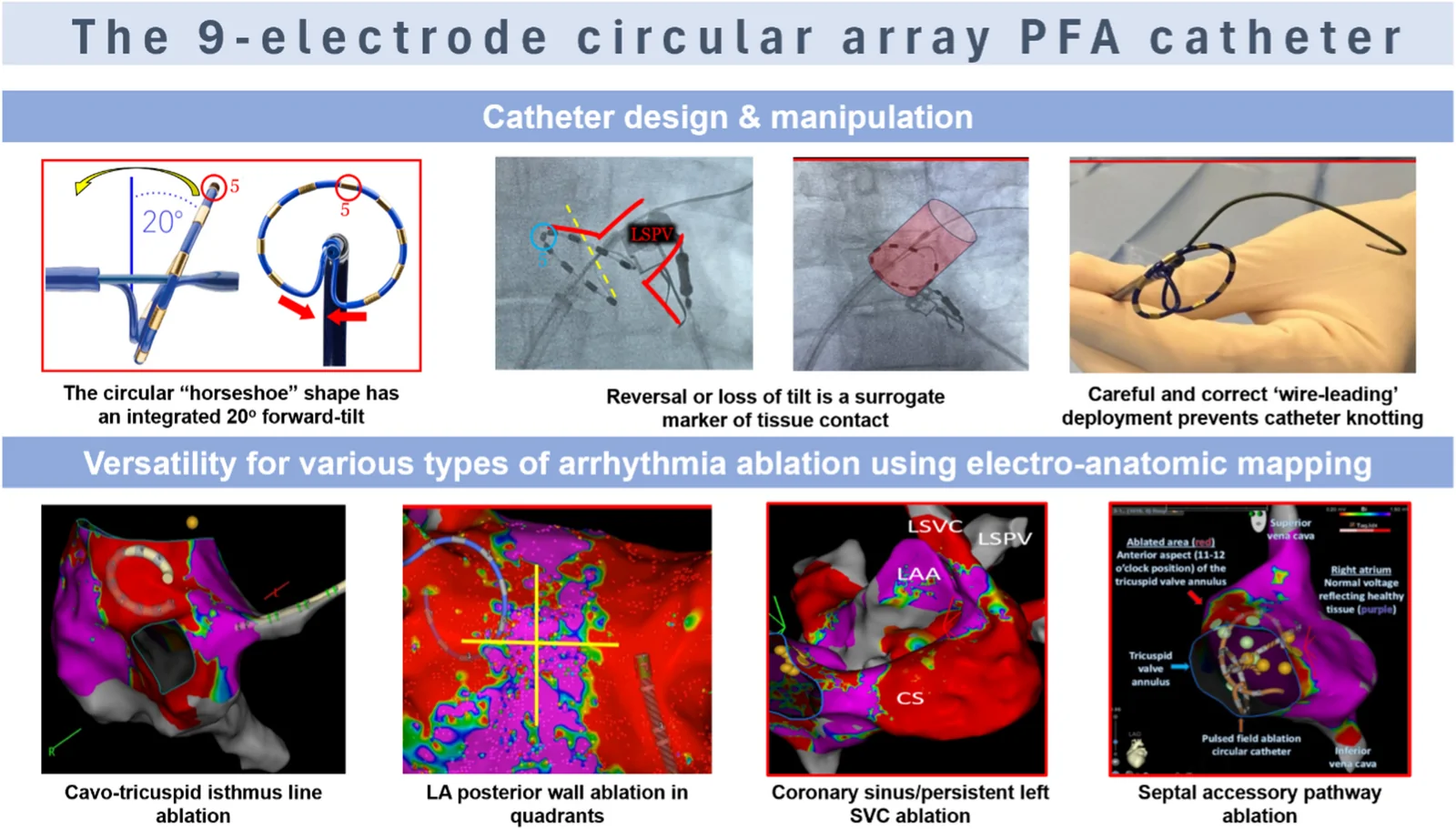

**Supplementary Information:**

The online version contains supplementary material available at https://doi.org/10.1007/s10840-026-02436-1.

## Capsule summary

This paper highlights tips and techniques for using the 9-electrode circular array PulseSelect™ Pulsed Field Ablation catheter, including setup and workflow for pulmonary vein isolation and other atrial ablation targets, as well as common pitfalls to avoid, in order to maximize safety, efficiency and efficacy.

**Supplementary Information:**

The online version contains supplementary material available at https://doi.org/10.1007/s10840-026-02436-1.

## Highlights

PulseSelect is a versatile pulsed field ablation catheter that is being increasingly adopted by electrophysiologists.

Its forward-tilt and circular/’horseshoe’-shaped design lends to tactile and visual contact feedback fluoroscopically.

It can also be used with electro-anatomic mapping for ablation to pulmonary veins and beyond.

We describe our tips and techniques to optimise ablation of various potential arrhythmia targets.

**Supplementary Information:**

The online version contains supplementary material available at https://doi.org/10.1007/s10840-026-02436-1.

## Introduction

Pulsed field ablation (PFA) is a cardiac ablation technology in which electrical fields are used to create microscopic pores in cardiomyocyte cell membranes, resulting in cell death [1]. Unlike traditional thermal ablation technologies (including radiofrequency and cryoballoon), PFA is more selective for cardiomyocytes, potentially reducing the risk of collateral damage to the oesophagus and phrenic nerve [2]. Several trials have shown the safety and efficacy of PFA [1, 3].

The PulseSelect™ ablation catheter (Medtronic Inc., Minneapolis, MN) is a 9-electrode circular PFA ablation catheter approved by regulatory agencies in the USA, Europe, and Japan. Early evidence suggests a favourable safety profile and comparable efficacy to well-established ablation technologies [3, 4]. The catheter can be used for pulmonary vein isolation (PVI) [1], but can also be used for ablations beyond the pulmonary veins. In this article, we present our real-world experience with the PulseSelect catheter to optimise its use for PVI and beyond.

## PulseSelect™ catheter design

**Fig. 1:**
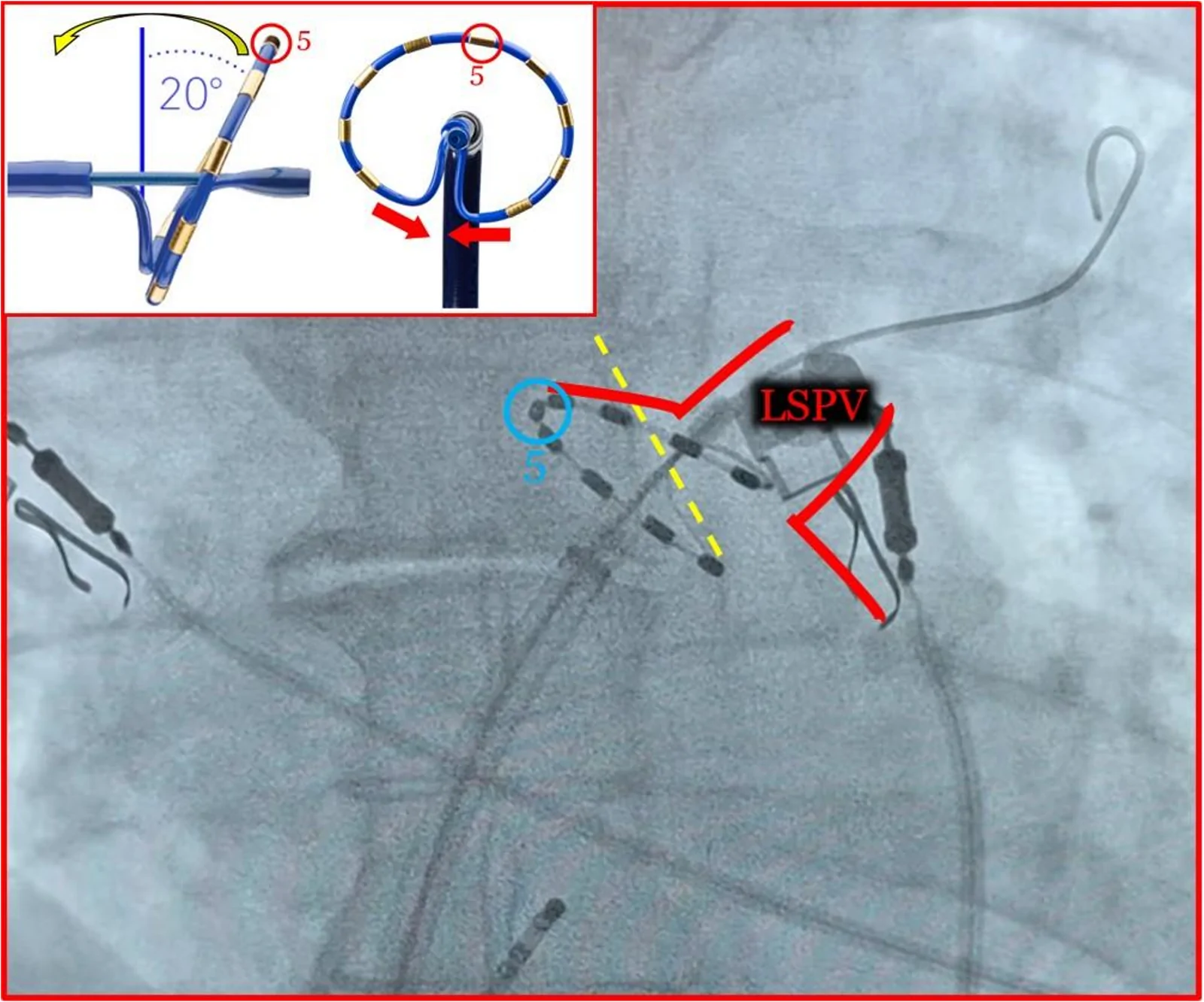
Electrode 5 (circled) is the centre of the energy application and projects furthest forwards. Loss or inversion of the 20° forward tilt (yellow arrow; yellow dotted line) serves as a surrogate marker of tissue contact when electrode 5 (highlighted in blue) contacts the myocardium. The sheath is purposefully misaligned with respect to the pulmonary vein to facilitate antral ablation (see text below). LSPV, left superior pulmonary vein

The catheter is composed of 9 electrodes arranged in a semicircular flexible array, surrounding an eccentrically shaped shaft that is closer to electrodes 1 and 9 and furthest from electrode 5. This array is tilted forward by 20 degrees, with electrode 5 at the most anterior position. Although the PulseSelect™ catheter is commonly described as circular, it is actually a forward-tilted, flexible horseshoe-shaped catheter, as no energy is delivered between electrodes 1 and 9. This design has several practical implications (Fig. 1).

Firstly, although PFA was initially thought to be less dependent on tissue contact, current understanding has reinstated its importance, given the observation of reversible myocardial stunning with recovery of ablated tissue within hours. This renders attenuation of intracardiac electrical signals immediately following energy delivery an unreliable marker of lesion durability. Therefore, tissue contact should be optimised to ensure durable ablation lesions have been delivered [5].

At baseline, electrode 5 is centrally located and projects furthest forward. When performing an application, electrode 5 should ideally face the critical target area, for example a conduction gap in an ablation line, a critical isthmus, or a specific side of the myocardium around a pulmonary vein (PV). The 20° forward-tilt can be used as a surrogate marker of tissue contact, as this angle is reduced or even inverted when electrode 5 is in contact with cardiac tissue (Fig. 1). It is also useful for identifying the ostium of the pulmonary veins, as the space between electrodes 1 and 9 narrows when the catheter is pushed inside the vein.

**Fig. 2:**
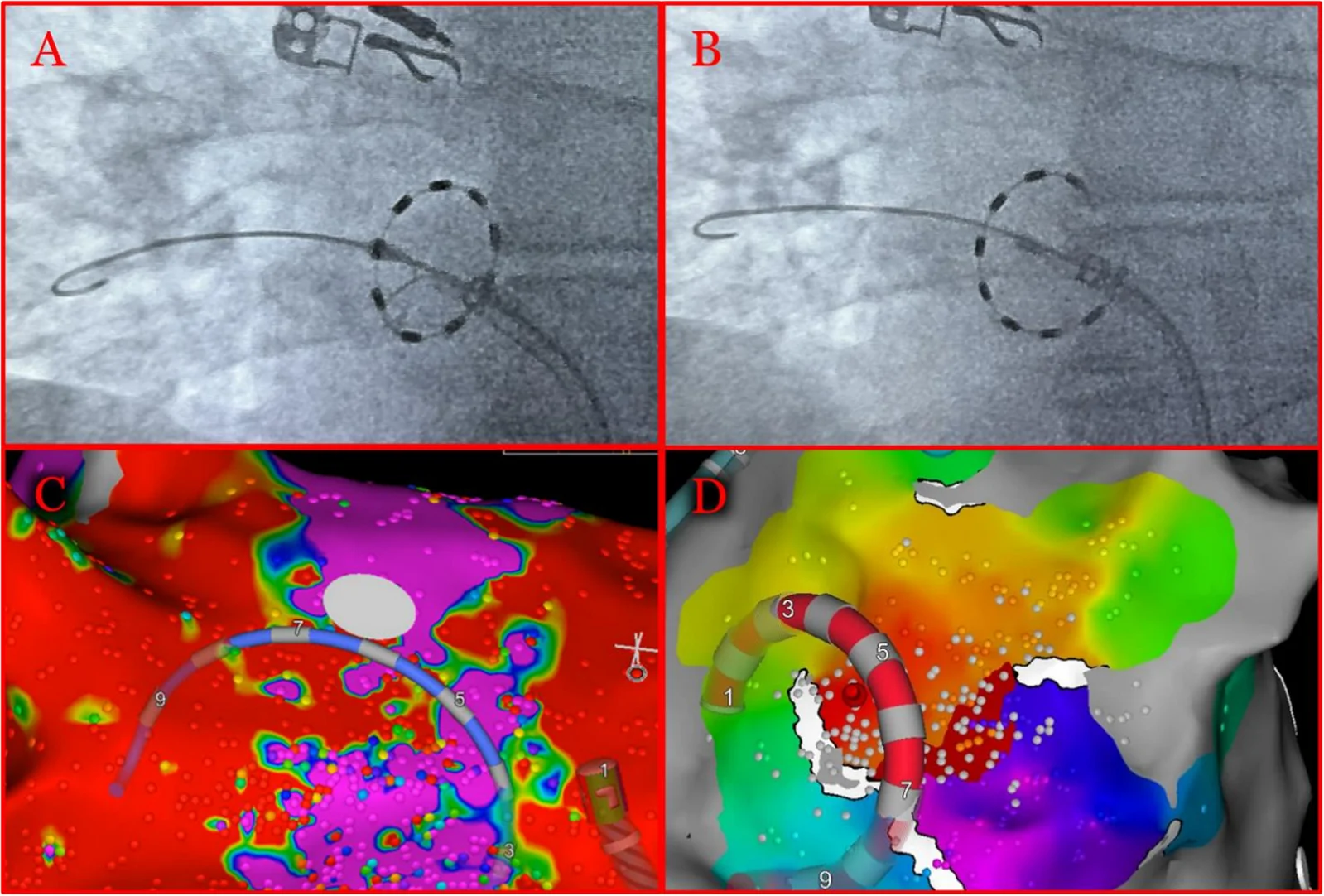
The flexibility of the PulseSelect catheter allows different ablation footprints, including circular (panel **A**), C-shaped (panel **B**), linear (panel **C**), and wide focal (panel **D**) configurations

The flexibility of the PulseSelect™ catheter allows different ablation patterns. Within tubular structures such as PVs, it can produce an almost circular ablation pattern. However, when retracted from the PV with the guidewire left inside the vein, the catheter can be advanced in a deliberately misaligned position to contact the antral atrial wall, thereby creating C-shaped antral lesions. Semilunar ablations can also be performed by anchoring electrode 5 to the wall and prolapsing the remaining electrodes forward. This manoeuvre is useful for ablating the left atrial posterior wall (LAPW), and is achieved by anchoring to the LAPW and advancing the catheter with clockwise rotation of the deflectable sheath. Similarly, it can be used to ablate the anterior aspect of the left superior PV (LSPV) by anchoring in the left atrial (LA) ridge and advancing the catheter with counterclockwise rotation of the deflectable sheath. Even linear ablation can be performed if the deflectable sheath is parallel to the myocardial wall, with electrode 5 pointing towards the target area, while rotating or adjusting sheath deflection as needed (Fig. 2).

The use of contact sensing using the PulseSelect™ catheter has been evaluated using a new impedance-based tissue proximity indicator that measures and displays real-time impedance changes at each catheter electrode on this circular multi-electrode PFA system. Initial data has demonstrated good performance of local impedance recordings for assessing catheter-tissue contact with this circular and visual feedback from the proximity indicator was able to increase the number of electrodes in close proximity to tissue during energy delivery [6].

## Catheter preparation

The FlexCath™ Contour deflectable sheath (Medtronic) is recommended for use with the PulseSelect™ catheter. Since the catheter has a three-dimensional configuration in its baseline working position and is linear when inside the sheath, correct deployment should be performed progressively with the guidewire secured inside a pulmonary vein (PV) (see Video 1 in the Supplementary Material for correct deployment and retrieval). Ablation is generally performed in its circular configuration, with the exception of the cavotricuspid isthmus (CTI), where it can be performed with the catheter in its sigmoid, stretched, or circular configuration (see below).

## Ablation at different cardiac locations

### Pulmonary vein isolation

A systematic workflow improves procedural efficiency, facilitates teaching, and enhances reproducibility. The following approach represents our preferred workflow, although alternative manoeuvres may achieve equivalent results. The recommended approach is to deliver at least eight applications per vein: four ostial and four antral. After gaining extensive experience with the PulseSelect™ system, our stand-alone PVI procedures, performed under fluoroscopic guidance alone without electro-anatomic mapping (EAM), are typically completed in 54 ± 7 min, with a fluoroscopy time of 12 ± 2 min, and a mean of 56 ± 3 PFA applications, corresponding to approximately four ostial and ten antral applications per pulmonary vein. These values reflect our current practice and may vary according to patient anatomy, operator experience, and the use of EAM.

The ostium corresponds to the junction between the tubular PV and the atrial wall, and contrast venography can help visualize this fluoroscopically. The antrum, however, has no universally accepted anatomical boundary, and its extent may therefore vary between operators. To differentiate ostial from antral ablation fluoroscopically, the J-tipped guidewire should first be secured inside the PV. The catheter is then advanced in its maximally deployed circular configuration over the guidewire until it reaches the atrial wall, identified by the loss of the forward tilt of the catheter. Further advancement into the vein produces a characteristic ‘jump’ and disappearance of the space between electrodes 1 and 9, both readily visible on fluoroscopy.

Although the PulseSelect™ system was initially designed as a fluoroscopically guided single-shot ablation system for PVI, operators have found that it can be readily visualized using available EAM systems, such as CARTO 3 (Biosense Webster Inc., Diamond Bar, CA) and EnSite X (Abbott, Abbott Park, IL). Initial maps are usually generated using each system’s proprietary high-definition mapping catheter, but it is also possible to map using the PulseSelect™ catheter itself in non-magnetic systems such as EnSite X. In our experience, EAM is particularly useful for minimizing fluoroscopy, as it improves visualization of the desired ablation level and important structures such as the left atrial appendage. A combination of EAM, fluoroscopy, and intracardiac electrograms can therefore be used to maximize accuracy. Although there are no standard recommendations, a personalized sequence for different catheter positions enhances efficiency and facilitates rapid understanding during proctorship. Initial applications can be ostial or antral, according to operator preference, but both GB and FO (present authors) prefer to perform ostial ablations before antral ablations.

As previously indicated, catheter orientation is determined by the position of the central No. 5 electrode. One technique is to rotate the catheter clockwise in the left PVs and counterclockwise in the right PVs using quarter-turns, progressing from superior to anterior, then anterior to inferior, inferior to posterior, and posterior to superior again. An alternative is to perform application sequences that ‘jump’ from superior to inferior and from anterior to posterior (half-turns instead of quarter-turns), thereby facilitating visual recognition of clearly different fluoroscopic positions.

Before rotation, it is advisable to slightly withdraw the catheter, rotate, and re-advance it to ensure that it detaches from the atrial wall, rotates freely, and then regains tissue contact in the new position (see Video 2 in the Supplementary Material). For ostial ablations, the deflectable sheath should be aligned with the centre of the pulmonary vein (PV), whereas for antral ablations it should be deliberately misaligned to direct the catheter towards the adjacent atrial wall (Fig. 1). The manoeuvres required to achieve antral ablation vary according to the target vein, but the underlying principles remain the same. The LSPV provides a useful example. For superior antral ablation, the sheath should be undeflected and the catheter advanced until it contacts the left atrial (LA) roof. Excessive contact should be avoided, as this may result in repeat ostial rather than antral ablation. For inferior antral ablation, electrode No. 5 is first oriented inferiorly, the sheath is deflected, and the catheter re-advanced until it reaches the LA carina. Similarly, clockwise rotation of the sheath with electrode No. 5 oriented posteriorly facilitates posterior antral applications. Finally, counterclockwise rotation of the sheath and advancement of electrode No. 5 against the LA ridge positions the catheter for ablation of the anterior LSPV antrum. The same principles apply to all pulmonary veins: electrode No. 5 should always be oriented towards the intended target, the sheath deliberately misaligned in the same direction, and the catheter gently advanced until adequate tissue contact is achieved.

**Fig. 3:**
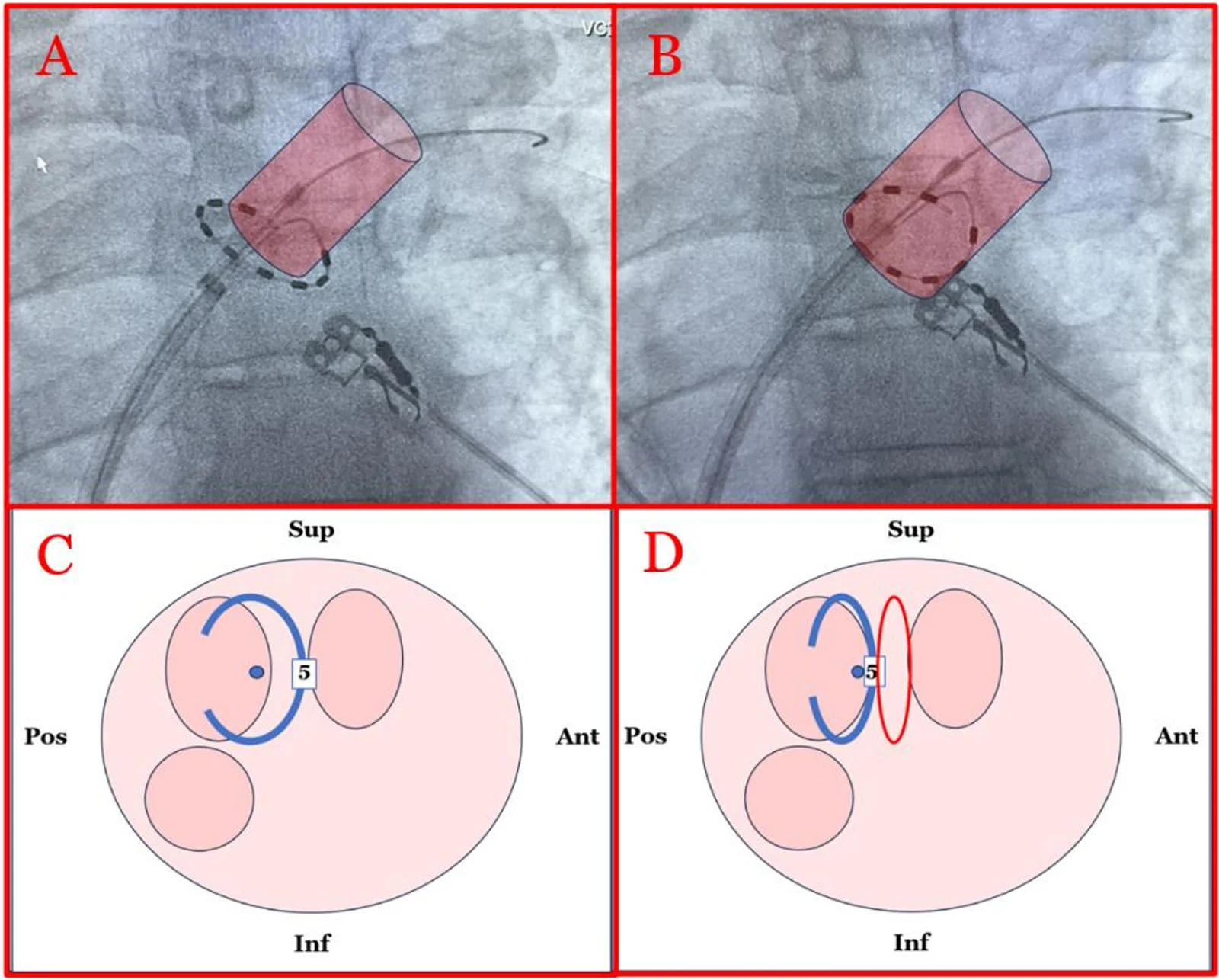
Two different fluoroscopic approaches for anterior ablation of the LSPV are shown. If the sheath is rotated and then the catheter is advanced, the left atrial ridge itself is ablated (panel **A**). If the catheter is advanced first and then the sheath is rotated afterwards, the inner anterior face of the vein is ablated (panel **B**). The former could be more appropriate for thicker/broader ridges, while the latter for a narrower ridge. In panels (**C**) and (**D**), a diagram of both techniques from a left anterior oblique endocardial view can be seen. Sup, superior; Pos, posterior; Ant, anterior and Inf, inferior

When ablating the anterior aspect of the LSPV, additional manoeuvres may be useful to optimise catheter contact at the LA ridge. In patients with a broad, flat LA ridge, directing the catheter towards the ridge summit may facilitate a more antral lesion set (Fig. 3, panels A and C). The potential drawback of this approach is that a narrow ridge may leave a small gap in lesion continuity. In such cases, the catheter can first be advanced into the LSPV, followed by advancement of the sheath with firm counterclockwise rotation, causing catheter prolapse and directing contact towards the anterior inner aspect of the LSPV, similar to the technique used for radiofrequency ablation of the LA ridge (Fig. 3, panels B and D). Given the low reported incidence of PV stenosis following PFA [2], these two complementary manoeuvres can therefore be selected according to LA ridge anatomy, or if desired, performed sequentially in all cases to maximise lesion continuity across the ridge.

When using EAM, the manoeuvres are largely unchanged, although lesion placement can be individualised while reducing fluoroscopy exposure. EAM also allows previously treated areas to be marked. One option is to create catheter-position ‘shadows’ before each application, although these may become too numerous and impair visualisation. An alternative is to tag only the position of electrode No. 5 before each application, allowing treated areas to be estimated without obscuring the map.

For PVI confirmation, the catheter can be used either in its fully deployed circular configuration or in a slightly stretched sigmoid configuration. In the latter, counterclockwise rotation facilitates catheter entry into the PV. Because the electrodes are 3 mm in diameter and separated by 3.75 mm, far-field signals are common in the left PVs. Baseline electrograms should therefore be recorded before ablation to facilitate comparison with post-ablation recordings. When using EAM, pre- and post-ablation LA maps can also be acquired for comparison.

### Posterior wall isolation (PWI) and LA roof line ablation

**Fig. 4:**
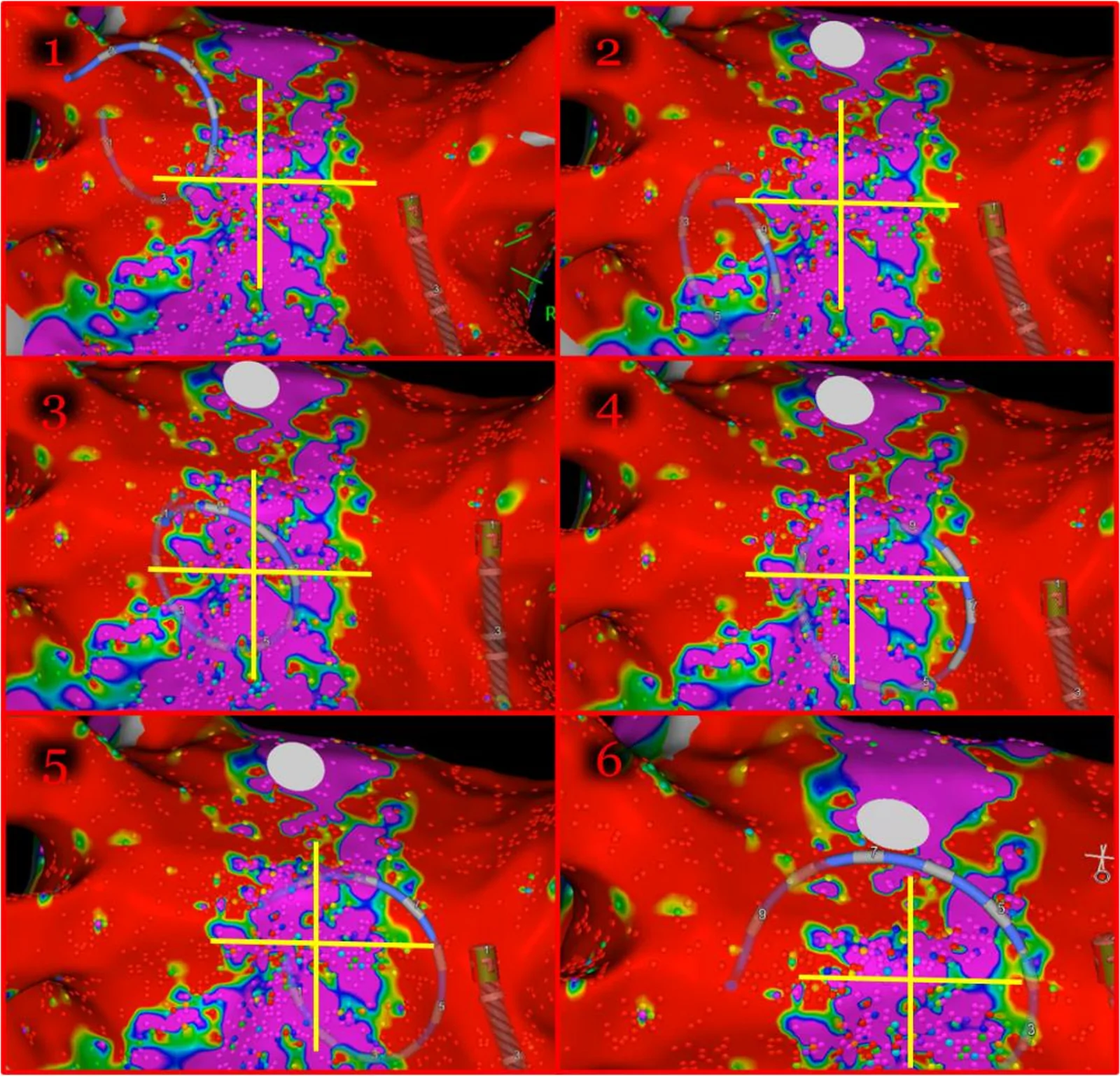
Posterior wall box isolation using 10 applications. During PVI, additional posterior antral applications from each PV may leave a narrow posterior corridor. Dividing this area into four quadrants (yellow lines) and delivering at least one application per quadrant (panels 1, 2, 4, and 5), together with one application for the inferior line (panel 3) and one for the roof line (panel 6), may achieve effective PWI

The PulseSelect™ catheter is well suited for ablation beyond the PVs, particularly for posterior wall isolation (PWI). While there is no single approach, we propose a systematic minimum protocol comprising ten applications, with additional applications added according to left atrial anatomy and the desired lesion set (the complete manoeuvre is shown in Fig. 4 and Video 3 in the Supplementary Material). The aim is to progress systematically across the posterior wall, ensuring overlap between adjacent applications while maintaining optimal tissue contact throughout the procedure. The first step is to consolidate PVI with wide posterior antral applications. This can be achieved by adding one additional posterior antral application for each PV to the standard PVI protocol. PWI can then be completed by delivering at least one application to each quadrant of the posterior wall, together with one application to the inferior line and one to the roof line.

### Cavo-tricuspid isthmus (CTI) ablation

**Fig. 5:**
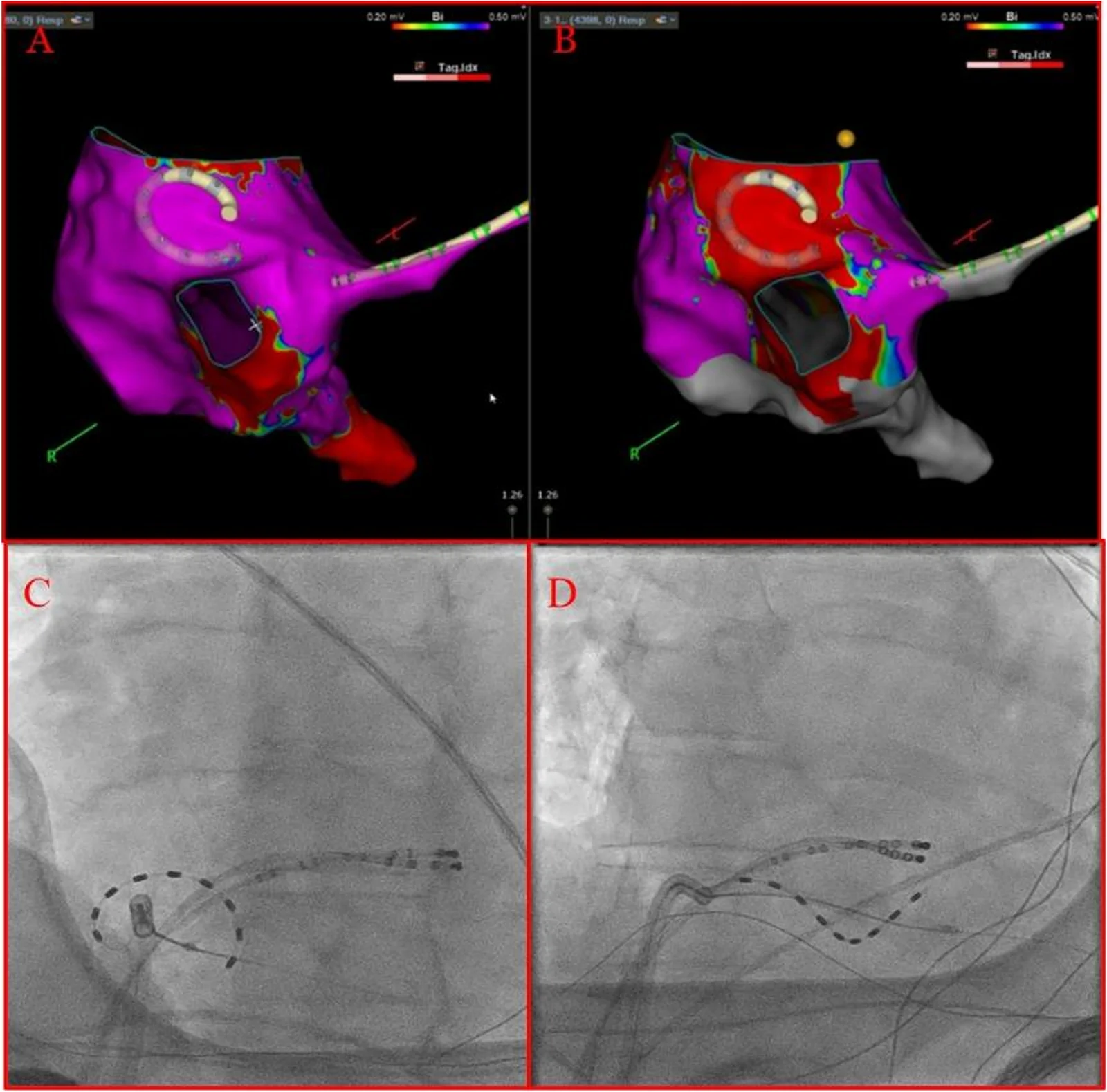
Pre-ablation (panel **A**) and post-ablation (panel **B**) electroanatomical maps (EAMs) of the cavotricuspid isthmus (CTI) in the left anterior oblique projection. A catheter-position ‘shadow’ corresponding to the first PulseSelect application is shown. CTI ablation was performed with the catheter in its circular configuration using six applications, resulting in a broad, homogeneous ablation line. Panels **C** and **D** show left anterior oblique and right anterior oblique fluoroscopic views, respectively, of CTI ablation performed with the catheter in a stretched sigmoid configuration, with four or five electrodes protruding beyond the sheath to form a linear array along the CTI and the guidewire positioned in the right ventricle

CTI ablation can be performed using two different approaches. With the catheter in its circular configuration, lateral CTI ablation is performed at the 7 o’clock position in the left anterior oblique projection to avoid para-septal structures (Fig. 5A and B). This approach creates a broad ablation line, typically requiring 4–8 applications, with a homogeneous lesion set and a clear transition to untreated tissue. Alternatively, the guidewire can be positioned in the right ventricle, the sheath deflected towards the tricuspid valve annulus, and the catheter stretched into a sigmoid configuration. The catheter is then positioned with the proximal electrodes inside and the distal electrodes outside the sheath, placing the distal electrodes along the CTI (Fig. 5C and D). This approach enables highly selective linear ablation but may be less effective because achieving consistent tissue contact can be challenging. We routinely administer 1 mg intravenous isosorbide dinitrate before ablation to reduce the risk of coronary vasospasm and, to date, have not observed vasospasm with either approach.

### Other ablation lines and targets

The PulseSelect™ catheter can also be used to create a variety of ablation lines beyond PVI, ranging from left atrial anterior linear ablation to ablation of atypical right atrial flutters, as previously reported [7, 8]. These non-standard applications should be tailored to the individual patient but generally follow the same principles of catheter manipulation: (1) keep the guidewire ahead of the catheter in a neutral position; (2) approach the target with the lateral aspect of the sheath to allow controlled catheter contact; (3) orient electrode No. 5 towards the critical isthmus or target site; (4) use slight catheter advancement as the final manoeuvre before PFA delivery to optimise tissue contact; and (5) maintain a safe distance from the conduction system.

**Fig. 6:**
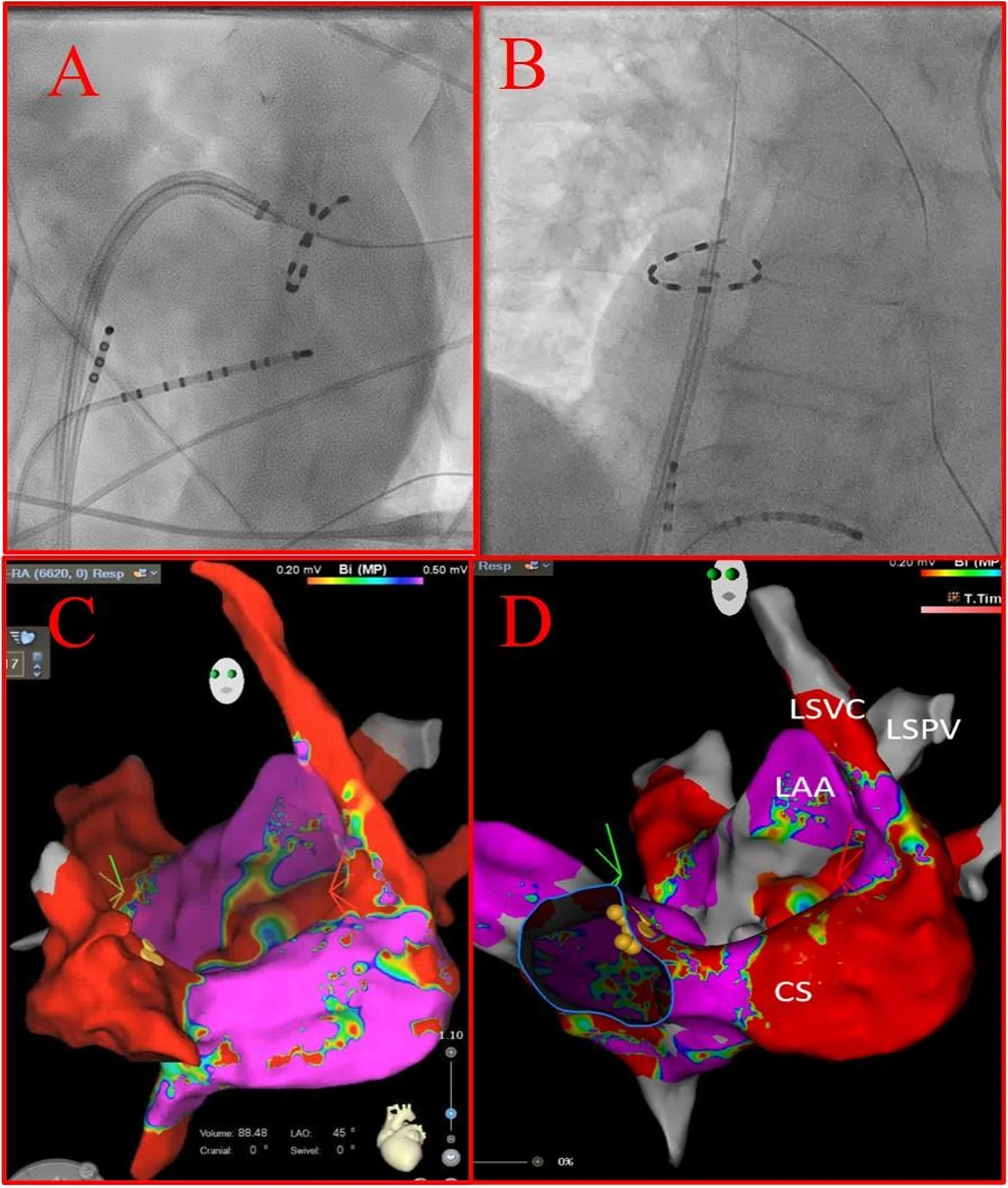
**A** Mitral valve isthmus ablation performed with the guidewire anchored in the left inferior pulmonary vein (LIPV), followed by gradual catheter advancement and withdrawal to deliver PFA along the desired ablation line. **B** Fluoroscopically guided superior vena cava (SVC) isolation with the PulseSelect catheter positioned at the SVC–right atrial (RA) junction. Panels **C** and **D** show pre-ablation and post-ablation electro-anatomic maps (EAMs) of a dilated coronary sinus in a patient with a persistent left-sided superior vena cava (LSVC). LSPV, left superior pulmonary vein; LAA, left atrial appendage; CS, coronary sinus

Mitral valve isthmus (MVI) ablation can be performed by positioning the guidewire in the left inferior PV and placing the PulseSelect catheter, in its circular configuration, across the MVI. Electrode No. 5 is then sequentially rotated to ensure adequate lesion overlap along the ablation line (Fig. 6A). Alternatively, the guidewire may be advanced into the left ventricular cavity. Superior vena cava (SVC) isolation, as well as ablation of a persistent left-sided SVC, can also be performed with the PulseSelect catheter. For conventional SVC isolation, the guidewire is advanced into the SVC and the catheter positioned at the SVC–right atrial junction. Sequential rotation of electrode No. 5 provides adequate lesion overlap around the vessel (Fig. 6B). We do not routinely perform phrenic nerve pacing and, to date, have not observed phrenic nerve palsy. For persistent left-sided SVC ablation, the guidewire is advanced into the left-sided SVC and the catheter positioned within the accessory chamber, away from septal structures, to ablate the coronary sinus ostium. In these cases, EAM is recommended to facilitate catheter navigation and lesion placement in complex anatomy (Fig. 6C and D).

**Fig. 7:**
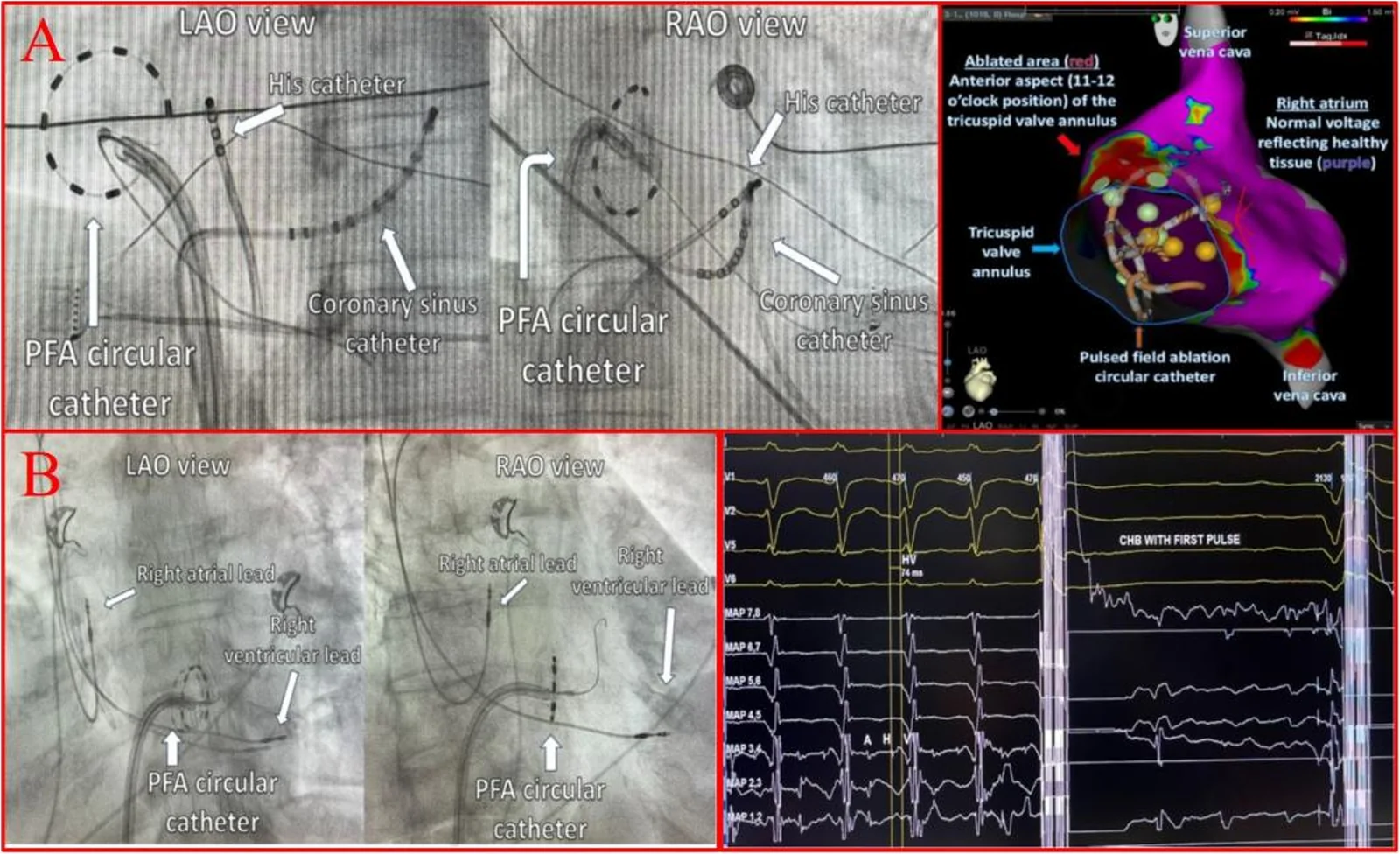
**A** Right-sided accessory pathway ablation performed under fluoroscopic and three-dimensional electroanatomical mapping (3D EAM) guidance, demonstrating acute procedural success with a low-voltage area in the target region on the post-ablation remap. **B** Fluoroscopically guided atrioventricular (AV) node ablation. Complete AV block was achieved with the first PFA application

We have also reported the use of the PulseSelect™ catheter for ablation of a right-sided accessory pathway [9]. This was performed in a young patient with recurrent supraventricular tachycardia following a previous unsuccessful thermal ablation procedure. The guidewire was positioned in the right ventricle (RV), with electrode No. 5 directed towards the accessory pathway. EAM was used to identify the His bundle and minimise the risk of atrioventricular (AV) block during ablation (Fig. 7A). The PulseSelect™ catheter has also been used for atrioventricular node (AVN) ablation, as previously reported [10]. The guidewire was again positioned in the RV and the catheter aligned with the AV node. Complete AV block was achieved with the first PFA application (Fig. 7B). In both cases, additional consolidation applications were delivered despite acute success with the first PFA application, with the aim of reducing the likelihood of recurrent accessory pathway or AV nodal conduction during follow-up.

## Things to avoid when using PulseSelect™

### Never manipulate the catheter without the guidewire ahead of the catheter tip

**Fig. 8:**
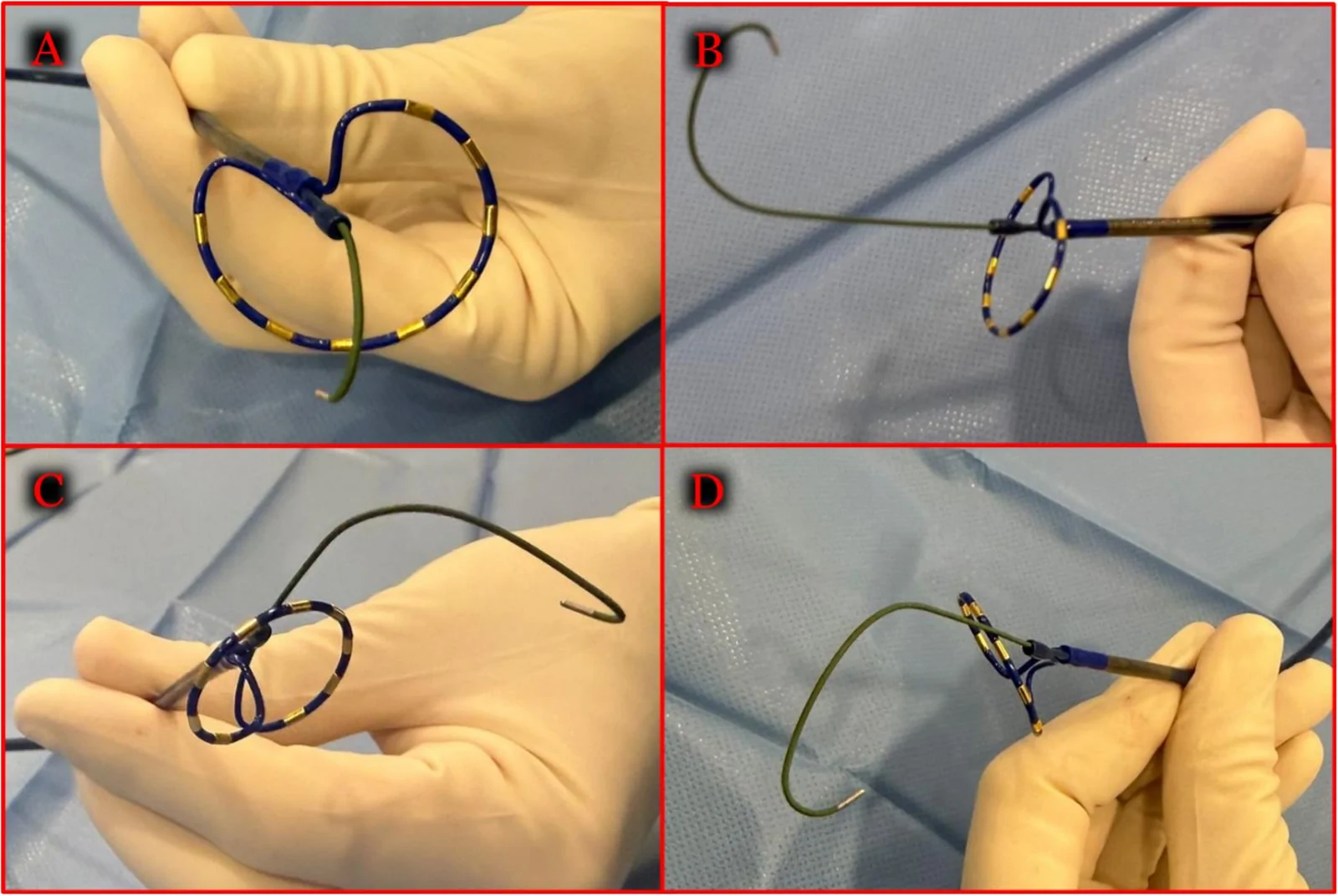
Correct catheter configuration in en face (panel **A**) and perpendicular (panel **B**) views. Although the catheter is neither circular nor symmetrical, the catheter tip, and therefore the guidewire, remain centrally positioned within the electrode array. Panels **C** and **D** show an abnormal configuration in which the guidewire is no longer central and the entire electrode array has moved to the same side of the catheter tip. On fluoroscopy, this appears as an inverted treble clef configuration and represents the initial stage of catheter knot formation. Withdrawing the catheter in this configuration may result in an irretrievable knot

This is a fundamental rule when using the PulseSelect™ catheter. Keeping the guidewire ahead of the catheter preserves its circular configuration by maintaining the tip at the centre of the electrode array. This helps prevent catheter knotting (Fig. 8), a complication that is largely avoidable but appears to be specific to the PulseSelect catheter. Three situations commonly predispose to knot formation.

The first occurs during initial catheter deployment in the LA. The guidewire should always be advanced well into a pulmonary vein (PV) before the catheter is progressively deployed into its circular configuration. A useful manoeuvre is to advance three electrodes beyond the sheath while retracting the handle slide-control by approximately one third. This retracts the guidewire lumen and distal tip, allowing the catheter to expand smoothly from its linear to its circular configuration.

The second situation may occur while moving from the left- to the right-sided PVs by clockwise sheath rotation. If electrode No. 5 remains anchored to the left atrial posterior wall (LAPW), continued clockwise rotation of the sheath may cause the entire electrode array to move to the same side of the catheter tip. If this is not recognised, subsequent guidewire advancement and continued catheter manipulation may result in catheter knotting. Directing electrode No. 5 inferiorly when moving from the left to the right PVs helps avoid this problem.

The third situation is encountered when cannulating the right inferior PV, particularly when the distance between the transseptal puncture site and the PV ostium is short. In these cases, it is preferable to keep the catheter within the sheath while advancing the guidewire into the vein. The catheter can then be deployed once guidewire position has been secured, avoiding forceful manoeuvres with the guidewire close to the catheter tip.

### Avoid deep PV energy applications

Antral and ostial ablation can be performed rapidly and effectively with the PulseSelect™ system, making deep energy applications within the pulmonary veins (PVs) unnecessary. These applications may induce ganglionated plexus and nerve stimulation [11] which, although unlikely to cause permanent injury, can prolong the procedure without providing additional benefit.

### Avoid delivering only the minimum number of ablations

During the learning curve, operators often follow the recommended protocol of eight applications per PV (four ostial and four antral). In our opinion, this should be considered the minimum rather than the target number of applications. From our experience, operators tend to deliver additional applications as confidence with the system increases, while procedure times continue to decrease. Importantly, no cases of severe or symptomatic haemolysis have been reported with the PulseSelect™ system to the best of our knowledge. Furthermore, PulseSelect™ has not been associated with greater haemolysis than cryoballoon PVI [12], and a recent registry also reported no significant haemolysis with this catheter [13]. Recent studies have also reported a trend towards a greater number of applications than originally recommended, which has been associated with a very high rate of durable pulmonary vein isolation during systematic invasive remapping procedures [14, 15]. Therefore, a modest increase in the number of applications to maximise durable PV isolation appears justified.

### Avoid underestimating the learning curve

Pulmonary vein isolation using PFA is often perceived as a straightforward procedure with little need for dedicated training. In our experience, however, successful and durable pulmonary vein isolation with the PulseSelect™ catheter depends on a thorough understanding of catheter geometry, tissue-contact principles, and manipulation techniques. We believe that procedural success with the PulseSelect™ catheter relies more on operator technique than with the pentaspline PFA catheter; an observation that is consistent with published learning-curve studies [16, 17]. This practical review was therefore developed to facilitate that learning process by describing the underlying principles of catheter manipulation rather than simply a sequence of procedural steps.

These considerations naturally raise the question of why the PulseSelect™ catheter should be selected in the first place. In our opinion, its principal advantage lies in the greater selectivity of lesion delivery. First, despite being a multipolar PFA catheter, it allows operators to target specific anatomical regions with greater precision, providing better control over lesion placement [7, 9, 18]. Second, previous studies have demonstrated a lower degree of haemolysis compared with pentaspline PFA systems [12]. Finally, the apparently lower incidence and severity of coronary vasospasm may be particularly advantageous when performing cavo-tricuspid and mitral isthmus ablation [19].

## Conclusions

The PulseSelect™ catheter combines a distinctive over-the-wire design with a forward-tilted flexible electrode array that provides considerable procedural versatility and, in our opinion, may offer advantages in terms of selective lesion delivery, reduced haemolysis, and a lower propensity for coronary vasospasm. Achieving these potential benefits, however, requires a thorough understanding of its geometry, tissue-contact principles, and manipulation techniques, which may help operators shorten the learning curve, optimise lesion delivery, and safely expand its applications beyond pulmonary vein isolation.

## Supplementary Information

Below is the link to the electronic supplementary material.

Supplementary Material 1 (Correct deployment and retrieval of the PulseSelect catheter.)

Supplementary Material 2 (Withdrawing the catheter before repositioning in order ensure free rotational movement and to optimise subsequent regain of tissue contact.)

Supplementary Material 3 (Catheter positions and manoeuvres to achieve Posterior Wall Isolation.)

## Data Availability

No datasets were generated or analysed during the current study.

## Abbreviations

PFA: Pulsed Field Ablation
PVI: Pulmonary Vein Isolation
PV: pulmonary vein
LSPV: left superior pulmonary vein
LA: left atrium
LAPW: left atrial posterior wall
PWI: posterior wall isolation
EAM: electro-anatomic mapping
SVC: superior vena cava
CTI: cavo-tricuspid isthmus
MVI: mitral valve isthmus
AVN: Atrio-ventricular node
